# Extraction of Bioactive Phenolics from Various Anthocyanin-Rich Plant Materials and Comparison of Their Heat Stability

**DOI:** 10.3390/molecules29225256

**Published:** 2024-11-06

**Authors:** Yanli Yu, Syyu Shiau, Weichen Pan, Yvette Yang

**Affiliations:** 1 Department of Food Nutrition and Safety, Sanda University, Shanghai 201209, China; ylyu@sandau.edu.cn (Y.Y.); yvette1101@126.com (Y.Y.); 2 Department of Food Science and Technology, Tajen University, Pingtung 90741, Taiwan; randy980625@gmail.com

**Keywords:** phytochemical, polyphenol, antioxidation, thermal degradation, extraction, kinetic

## Abstract

Butterfly pea flower (BPF), roselle calyx (RC), and grape skin (GS) are rich in bioactive phenolics with health benefits. Due to its simplicity, safety, and environmental friendliness, this study used water as a solvent to explore different extraction conditions in these plant materials and compared the heat stability of anthocyanins in the aqueous extracts. To maximize the total anthocyanins and polyphenols in the aqueous extracts, the powders of BPF, GS, and RC should be extracted for 30 min at 90 °C; 30 min and 120 min at 90 °C; and 30 min and 60 min at 60 °C, respectively. Among the tested plant materials, the content of total anthocyanins was RC > GS > BPF, while the total phenolic content was GS > BPF > RC. Anthocyanins of the aqueous extracts underwent rapid thermal degradation at high temperatures and high pH values. The thermal stability of anthocyanins in the materials was in the order: BPF > GS > RC. This is likely related to the types and structures of the anthocyanins such as the degree of acylation and glycosylation. The study demonstrates that hot water extraction is efficient and practical for these materials, yielding extracts suitable for food and nutraceutical applications.

## 1. Introduction

Anthocyanins are important natural water-soluble phenolic pigments of plants commonly found in vegetables, fruits, and cereals. In addition to displaying a variety of colors, they also possess numerous physiological benefits including antioxidant, anti-inflammatory, cardiovascular, hypoglycemic, and prebiotic effects [1,2,3]. More than 700 anthocyanins have been identified to date [4] and are composed of anthocyanidins and sugars, which are sometimes bound to aliphatic or aromatic organic acids [5]. Delphinidin, cyanidin, pelargonidin, malvidin, petunidin, and peonidin are the six most common anthocyanidins.

Due to the electron-deficient flavylium cation, anthocyanins are highly reactive in chemical reactions, which cause their structure to change, leading to fading or discoloration. The stability of anthocyanins is affected by their chemical structure and various environmental factors including pH, temperature, light, oxygen, metal ions, enzymes, and water activity [6,7]. The glycosylation of anthocyanidins generally leads to an increase in stability and water solubility and a decrease in antioxidation [8]. Acylated anthocyanins have a higher stability and antioxidant capacity than non-acylated anthocyanins because the former improve color stability via copigmentation and self-association reactions [5,7]. Heating at high temperatures causes the thermal degradation of anthocyanins, which generally fits well with a first-order reaction kinetic model [9]. Anthocyanins are stable in acidic conditions, but their degradation is often promoted in neutral and alkaline pH [10]. By utilizing the pH sensitivity and color changes of anthocyanins, pigment-based films can be used to visually monitor the freshness of protein-rich food products [7,11].

Both butterfly pea and roselle flowers are widely used as natural colorants in foods and beverages. Due to its rich content of bioactive anthocyanins, polyphenolics, phytosterols, and tocopherols, butterfly pea flower (BPF) has the ability to combat oxidative stress and promote overall wellness [12,13]. BPF contains a large amount of blue ternatins, which are polyacylated derivatives of delphinidin 3,3′,5′-triglucoside [14]. Roselle calyx (RC) contains bioactive compounds such as anthocyanins, organic acids, flavonoids, phenolic acids, and ascorbic acid and is known for its antibacterial, antioxidant, antihypertensive, antidiabetic, hepatoprotective, and nephroprotective effects [15,16]. Sambubiosides of both delphinidin and cyanidin are the main anthocyanins in RC extracts [17]. Grape skin (GS), a by-product of juice and wine production, is abundant in phytochemicals including anthocyanins, proanthocyanidins, flavonoids, resveratrol, stilbenoids, and dietary fiber, which give it potential as a functional food ingredient [18,19]. The variety of grape affects the types of anthocyanins in GS. The skin of black grape varieties has high levels of malvidin-3-O-glucoside, while red grape varieties primarily contain the glucosides of cyanidin and delphinidin [1,20]. Morata et al. indicated that bluish-red grapes contained more acylated anthocyanins than red-orange grapes such as malvidin-3-O-(6-O-p-coumaroyl)-glucoside, malvidin-3-O-(6-O-acetyl)-glucoside, and petunidin-3-O-(6-O-p-coumaroyl)-glucoside [21].

The choice of solvent for the extraction of bioactive substances plays a crucial role in determining their stability and extraction efficiency. Organic alcohol (methanol or ethanol) and acetone are often employed to extract anthocyanins from plant materials, as these solvents can penetrate the plant cell walls more effectively and dissolve bioactive components [1,22,23]. Recent reports [24,25,26] have highlighted the use of green extraction techniques such as ultrasound-assisted, microwave-assisted, high-pressure processing, ohmic heating, and supercritical fluid extraction. These green techniques offer benefits including shorter extraction times, lower extraction temperatures, and energy consumption as well as higher extraction yields. However, water as a solvent has several advantages: it is simple, non-toxic, inexpensive, safe, and environmentally friendly, making it more suitable for the food and nutraceutical industries [6,27,28]. However, there is still limited systematic research exploring the water extraction of biophenolics and the antioxidant properties of BPF, RC, and GS.

Powders of BPF, RC, and GS were prepared and used in this study. The aqueous extraction process was conducted at different temperatures (30–90 °C) and periods (30–120 min). The bioactive phenolics and antioxidant activities of the resulting aqueous extracts (i.e., butterfly pea flower extract (BPFE), roselle calyx extract (RCE), and grape skin extract (GSE)) were then determined. This research aimed to identify the optimal extraction conditions for these anthocyanin-rich plant materials. Additionally, traditional 60% ethanol, extracted at 40 °C for 30 min, was used as the solvent in the control group to compare with the effects of water extraction. Finally, the thermal stability of the anthocyanins in the extracts was examined under various heating temperatures (60–90 °C), heating times (0–5 h), and pH levels (2.5–5.5).

## 2. Results and Discussion

### 2.1. Bioactive Phenolics of Extracts

The pH values of the BPFE, GSE, and RCE prepared was 5.82–5.91, 4.74–4.79, and 2.65–2.71, respectively, and they were not obviously changed by various extraction conditions. The roselle extract had a strong tart flavor due to the high amounts (11.31%) of organic acids including malic acid, tartaric acid, citric acid, hibiscus acid, and hydroxycitric acid [15,17,29].

**Figure 1:**
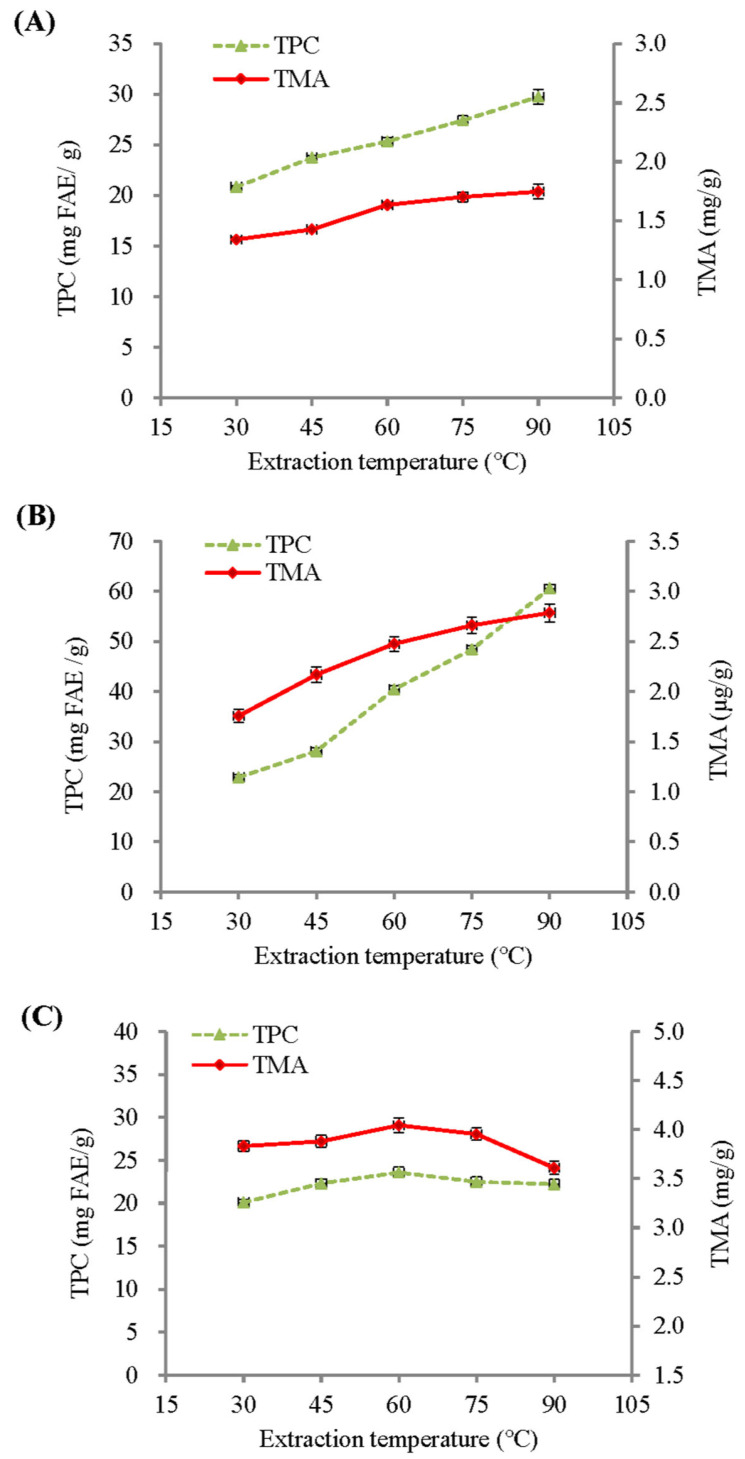
Influence of extraction temperature on the total monomeric anthocyanin (TMA) and total phenolic content (TPC) of aqueous extracts under a fixed 30-min extraction time: (**A**) BPFE; (**B**) GSE; (**C**) RCE.

Figure 1 shows the influence of extraction temperature on the total monomeric anthocyanin (TMA) and total phenolic content (TPC) of BPFE, GSE, and RCE. With a fixed extraction time of 30 min, the TMA and TPC of both the BPFE and GSE showed a significant increase as the extraction temperature was raised from 30 °C to 90 °C. Within this temperature range, the TMA and TPC of the BPFE were greatly increased by 30.24% and 42.79%, while GSE was increased by 58.55% and 164.94%, respectively. However, both the TMA and TPC of the RCE (Figure 1C) showed the highest values when extracted at 60 °C. The enhancement of these bioactive compounds with higher extraction temperatures was partly due to the increase in internal energy of the molecules, which resulted in greater molecular diffusivity and solubility. Furthermore, extraction at high temperature could influence the plant cell membrane structure and permeability, thus enhancing the extraction of cellular solutes by aqueous solvents. The thermal stability of bioactive compounds during extraction is also an important factor affecting extraction yield. The combined effects of these factors resulted in varying TMA and TPC extraction yields for the BPFE, GSE, and RCE at different extraction conditions.

Extraction temperature and time can influence the content of bioactive substances and their bioactivity. Table 1 lists the effect of extraction time on the TMA and TPC of the BPFE, GSE, and RCE. The TMAs of the BPFE and GSE extracted at 90 °C and RCE at 60 °C gradually decreased as the extraction time increased from 30 to 120 min, with their respective reductions reaching 8.53%, 20.31%, and 7.18% after 120 min of extraction. To acquire the highest TMA, the BPFP, GSP, and RCP should be extracted for 30–90 min at 90 °C, 30 min at 90 °C, and 30–60 min at 60 °C, respectively. The order of TMA values in the tested materials from highest to lowest was RCE (4.04 mg/g), GSE (2.79 mg/g), and BPFE (1.75 mg/g).

**Table 1:** Influence of extraction time on the bioactive phenolics and antioxidation activities of BPFE, GSE, and RCE.

Sample	Extraction Time (min)	TMA (mg/g)	TPC (mg FAE/g)	DPPH Scavenging Activity (mg TE/g)	Reducing Power (mg TE/g)
BPFE	30	1.75 ± 0.05 ^a^	29.73 ± 0.70 ^a^	26.51 ± 0.66 ^b^	33.84 ± 0.95 ^a^
BPFE	60	1.70 ± 0.05 ^ab^	30.50 ± 0.64 ^a^	27.04 ± 0.68 ^b^	34.07 ± 0.92 ^a^
BPFE	90	1.66 ± 0.05 ^ab^	31.09 ± 0.44 ^a^	27.74 ± 0.70 ^ab^	34.41 ± 0.96 ^a^
BPFE	120	1.59 ± 0.04 ^b^	31.10 ± 0.47 ^a^	28.42 ± 0.72 ^a^	34.78 ± 0.96 ^a^
GSE	30	2.79 ± 0.09 ^a^	60.55 ± 0.84 ^d^	109.96 ± 2.19 ^c^	111.01 ± 2.75 ^a^
GSE	60	2.56 ± 0.08 ^b^	65.01 ± 0.70 ^c^	112.81 ± 2.22 ^b^	112.14 ± 2.52 ^a^
GSE	90	2.49 ± 0.09 ^b^	67.51 ± 0.86 ^b^	120.32 ± 2.61 ^a^	113.86 ± 2.69 ^a^
GSE	120	2.22 ± 0.08 ^c^	69.18 ± 0.81 ^a^	123.32 ± 2.83 ^a^	115.61 ± 2.85 ^a^
RCE	30	4.04 ± 0.07 ^a^	23.58 ± 0.62 ^b^	35.78 ± 0.88 ^a^	41.93 ± 0.71 ^b^
RCE	60	3.95 ± 0.08 ^ab^	24.56 ± 0.69 ^ab^	35.87 ± 0.81 ^a^	47.10 ± 0.82 ^a^
RCE	90	3.87 ± 0.07 ^bc^	25.41 ± 0.50 ^a^	35.43 ± 0.88 ^a^	47.35 ± 0.78 ^a^
RCE	120	3.75 ± 0.06 ^c^	25.62 ± 0.57 ^a^	35.32 ± 0.82 ^a^	47.63 ± 0.75 ^a^

Values are means ± standard deviations (*n* = 3), and means with different letters in the same column and section are significantly different (*p* < 0.05). BPFE: butterfly pea flower extract; GSE: grape skin extract; RCE: roselle calyx extract.

Generally, a longer extraction time can enhance the extractability of a heat-tolerant substance enclosed within strong cell walls. Although the TPC of BPFE was insignificantly affected by extraction time (*p* > 0.05), the TPCs of both GSE and RCE significantly increased with extraction time, with ratios of 14.25% and 8.65%, respectively. Hence, the total polyphenols present in these materials may be relatively heat-stable. A longer extraction time was beneficial for the yield of total polyphenolics from these materials, especially GSP. This may be due to the tissue structure of the fruit peel being stronger or having a thicker cell wall compared to that of the fruit pulp and flowers [21]. Under the optimal extraction conditions, the TPC of the test materials followed the order: GSE (69.2 mg FAE/g, 120 min at 90 °C) > BPFE (30.0 mg FAE/g, 30–120 min at 90 °C) > RCE (25.4 mg FAE/g, 90 min at 60 °C).

Some studies on anthocyanin extraction using hot water as a solvent have indicated the optimal extraction conditions for plant materials. For butterfly pea flower, these conditions included 60 °C for 60 min [30], 80 °C for 30 min [14], 90 °C for 5 min [31], 100 °C for 5 min [32], and 100 °C for 30 min [33]. The variation in results could be attributed to factors such as the range of extraction temperatures, times and pressures tested, particle size of the samples, solid-to-solvent ratio, stirring conditions, and the analytical methods used for anthocyanins [3,27].

Our TPC results for butterfly pea flower aligned with previous studies (41–69 mg GAE/g extract) [34,35]. However, Ratha et al. reported a higher TPC of 160 mg GAE/g extract for aqueous extracts produced at 90 °C for 30 min [36]. Escher et al. found no significant differences in TPC between low and high extraction temperatures (11.7–60 °C) across different times (15, 30, 45 min) [34]. Phenolic compounds identified in the extract included sinapic, ellagic, 2,4-dihydroxybenzoic, protocatechuic, gallic, and caffeic acids [34,36].

The optimal extraction conditions for anthocyanins from roselle in this study were in an agreement with those in the study [3], which reported that extraction temperatures above 60 °C were unfavorable for roselle, as the anthocyanin yield significantly decreased with longer extraction times (60–300 min) at 80–90 °C. However, Wong et al. indicated that the optimal extraction condition for hibiscus anthocyanins was 60 °C hot water extraction for 3.5 h by response surface methodology [16]. Under this condition, the amount of anthocyanins obtained was 11% higher compared to using a blended method of 100 °C boiling water (non-isothermal extraction). Sindi et al. reported that when using water at different temperatures to extract roselle powder for 10 min, the total anthocyanin content was as follows: 100 °C > 50 °C > 25 °C [37]. The highest total anthocyanin content in roselle calyx was achieved using 70% ethanol for 35 min at 60 °C with ultrasound assistance [1].

The TPC of roselle calyx ranges from 0.78 to 291.78 mg GAE/g, depending on the cultivar and extraction conditions [38]. Using water extraction at three different temperatures for 10 min, the TPC of roselle decreased in the following order: 50 °C > 100 °C > 25 °C [37]. Optimal extraction with 70% ethanol at 60 °C for 35 min yielded 32.67 mg GAE/g [1]. Caffeic, chlorogenic, and p-coumaric acids, along with catechin, quercetin, and hesperidin, were identified in the methanolic roselle extracts by HPLC analysis [39].

Grape pomace is a good source of polyphenolic compounds, with a TPC ranging from 69 to 104 mg GAE/g residue using 80% ethanol extraction. The identified polyphenolic compounds included syringic acid, catechin, gallic acid, caffeic acid, and epicatechin [18]. To our knowledge, there is limited research on the aqueous extraction of bioactive phenolics from grape skins. Higher water extraction temperatures (100 °C and 70 °C) resulted in higher total anthocyanin and polyphenol contents in grape skins compared to room temperature extraction [40]. Moreover, the total anthocyanins and polyphenols extracted with boiling water were lower (67% and 64%, respectively) than with 70% ethanol. Despite this, heated water extraction was as effective as, or better than, 70% ethanol for recovering individual hydrophilic polyphenols. The optimal temperatures for pressurized liquid extraction of total anthocyanins (monoglucosides plus acylated forms) from grape skins were 80–100 °C with acidified water [41]. As the extraction temperature increased from 20 °C to 80 °C, the total anthocyanin yield rose significantly, while the ratio of monoglucosides to acylated anthocyanins decreased from 6.63 to 3.90. Liazid et al. reported that the anthocyanins in red grape skins were stable at temperatures up to 100 °C during pressurized liquid extraction. However, degradation levels of 40% to 50% were observed at 125 °C [24].

The type of solvent affects the extraction yield and composition of the bioactive compounds in plant materials since different solvents have varying polarities. The polarity of common solvents, from highest to lowest, is as follows: water > methanol > ethanol > acetone > ethyl acetate > hexane. In this study, the TMA and TPC of BPF powders extracted using the conventional 60% ethanol method at 40 °C for 30 min were 1.98 ± 0.05 mg/g and 33.56 ± 0.54 mg/g, respectively. Under the optimal conditions shown in Table 1 (90 °C for 30–120 min), the TMA and TPC of BPFE were 11.6% and 7.3–11.4% lower, respectively, compared to the ethanol extraction. These findings are consistent with the study of Ludin et al. [22], who found that absolute ethanol extracted more anthocyanins from butterfly pea flowers than water or nonpolar solvents. However, Netravati et al. observed similar TPC levels in aqueous and 50% ethanolic extracts of butterfly pea petals at 45 °C for 45 min [42].

For the RC powders, the TMA and TPC extracted using the conventional ethanol method in this study were 3.77 ± 0.08 mg/g and 25.02 ± 0.62 mg/g, respectively. The anthocyanin content was 6.7% lower than that of the aqueous extract (4.04 mg/g) obtained after 30 min at 60 °C, as shown in Table 1, while the TPC levels obtained by both the ethanol and aqueous methods were comparable. The variations in total anthocyanins and polyphenols between the two solvents in RC and BPF were inconsistent. This is likely because the main anthocyanins in roselle are anthocyanin glycosides, which are highly polar and more water-soluble, making aqueous extraction more suitable. In contrast, the primary anthocyanins in butterfly pea flowers are acylated anthocyanins, which are slightly less polar, making ethanol a more effective solvent. There have been reports on studies showing that the water extraction of anthocyanins from roselle is more effective than alcohol extraction. Sindi et al. reported that the aqueous extract of roselle flowers contained significantly higher total anthocyanin levels than the methanolic extract at the same extraction temperatures for 10 min [37]. Aryanti et al. reported that roselle extracted with distilled water yielded significantly higher total anthocyanin contents than when extracted with ethanol at room temperature for 24 h [2]. However, the total anthocyanin and phenolic contents of roselle, extracted using water at room temperature for 24 h, or by blending with boiling water and allowing it to stand for 5 min, were comparable to those extracted using 80% ethanol at 25 °C for 24 h or 2 h, respectively [17,39].

### 2.2. Antioxidation Capacity of Extracts

**Figure 2:**
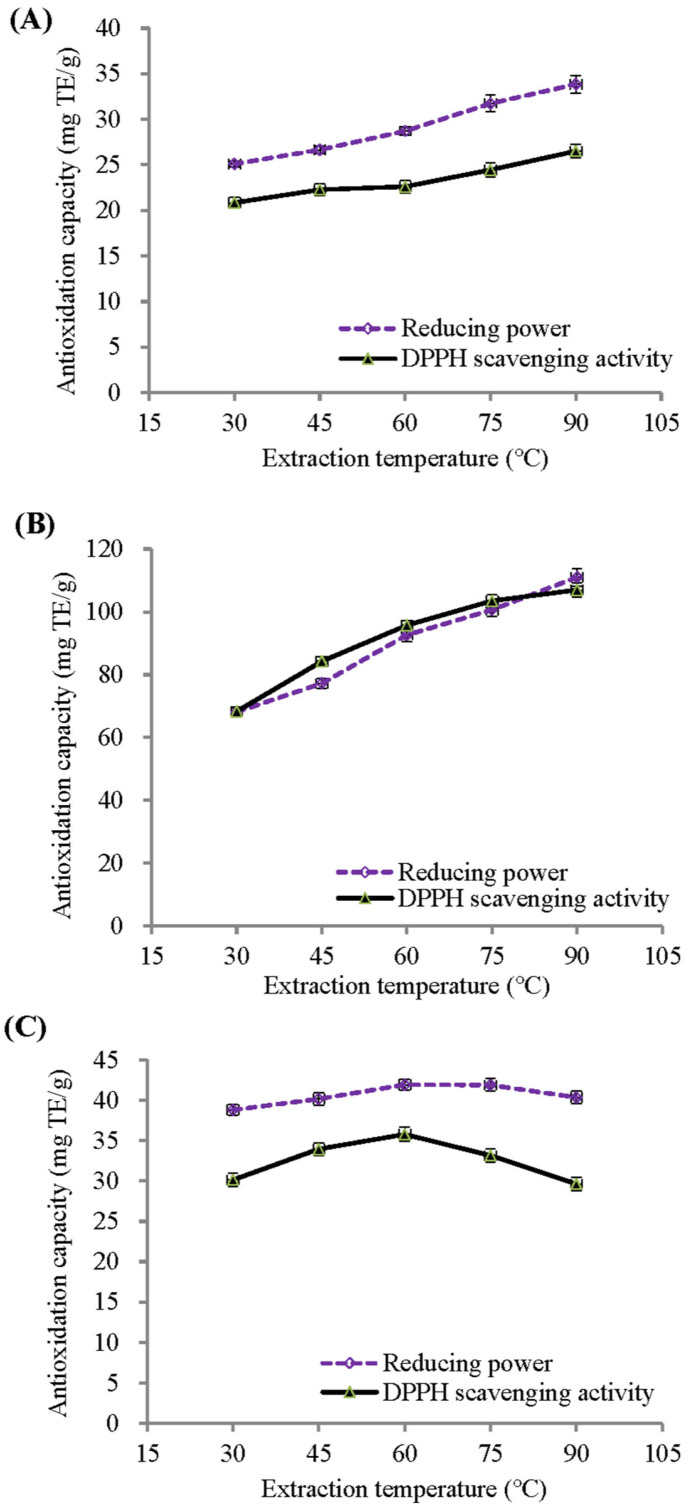
Influence of extraction temperature on the 2,2-diphenyl-1-picrylhydrazy (DPPH) radical scavenging activity and reducing power of aqueous extracts under a fixed 30-min extraction time: (**A**) BPFE; (**B**) GSE; (**C**) RCE.

**Figure 3:**
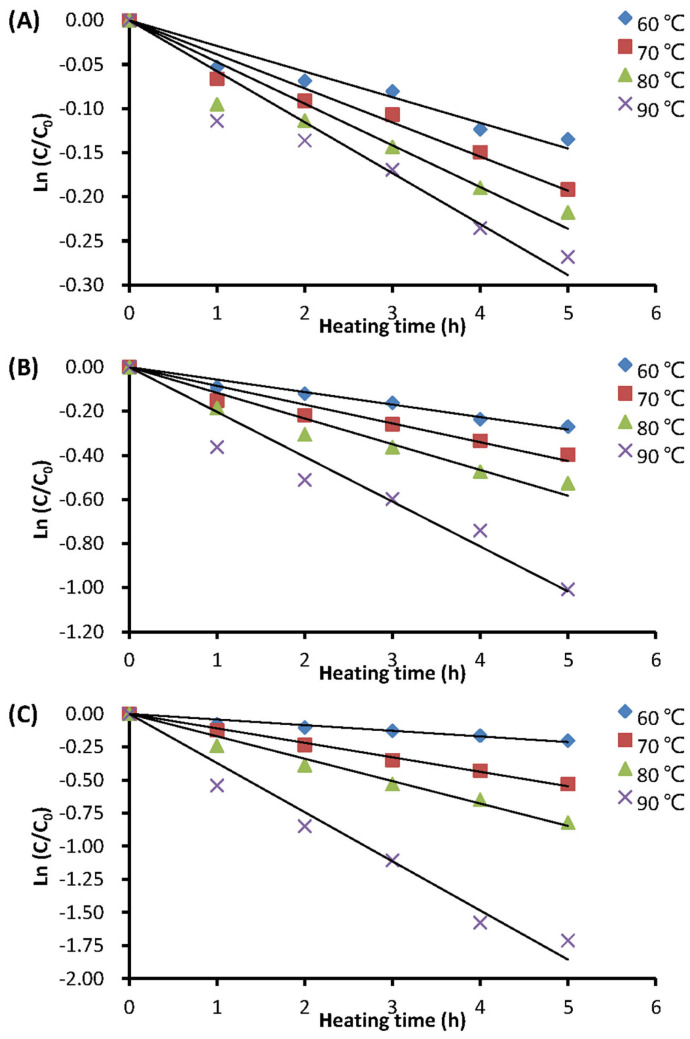
Thermal degradation curves of anthocyanins at pH 5.5 in different plant extracts: (**A**) BPFE; (**B**) GSE; (**C**) RCE: C_0_ is the initial monomeric anthocyanin content; C is the monomeric anthocyanin content after a certain heating time at a given temperature.

Similar to the TMA and TPC shown in Figure 1, the 2,2-diphenyl-1-picrylhydrazy (DPPH) scavenging activity and reducing power of both the BPFE and GSE significantly increased as the extraction temperature was raised from 30 °C to 90 °C (Figure 2). Within this temperature range, the DPPH scavenging activity and reducing power of the BPFE increased by 27.18% and 34.82%, respectively, while the GSE increased by 56.65% and 62.93%, respectively. However, the antioxidant capacity of RCE (Figure 3C) was highest when extracted at 60 °C, with its DPPH scavenging activity and reducing power increasing by 18.68% and 8.04%, respectively. However, the antioxidant capacity of RCE (Figure 3C) was highest when extracted at 60 °C, with its DPPH scavenging activity and reducing power increasing by 18.68% and 8.04%, respectively.

As the extraction time increased from 30 to 120 min at 90 °C (Table 1), the DPPH scavenging activity of BPFE and GSE increased by 7.21% and 15.29%, respectively. However, the DPPH scavenging activity of the RCE was not obviously affected by the extraction time at 60 °C. Conversely, the extraction time did not significantly influence the reducing power of the BPFE and GSE (*p* > 0.05), while the reducing power of the RCE increased by 13.59%. To acquire the optimal DPPH scavenging activity and reducing power, the BPFP, GSP, and RCP could be extracted for 120 and 30 min at 90 °C, 90 min and 30 min at 90 °C, and 30 and 60 min at 60 °C, respectively. The order of two antioxidation activities in the tested extracts, from highest to lowest, was GSE, RCE, and BPFE.

The antioxidant capacity of various anthocyanidins increases with the number of hydroxyl groups on their B-ring and decreases with an increase in methoxy groups. The antioxidant activity of the six anthocyanidins decreased in the following order: delphinidin > petunidin > cyanidin > malvidin > pelargonidin > peonidin [37,43]. Ternatins in BPFE had the fewest hydroxyl groups on the B-ring, as many had been substituted with sugars and organic acids. This may have led to BPFE showing the lowest antioxidant activity among the various plant extracts in Table 1.

Generally, substances rich in bioactive phenolics exhibit good antioxidant capacity [37,44]. In the BPFE, there were significantly positive correlations (*p* < 0.05 or 0.01) between TMA as well as TPC and both the DPPH scavenging activity and reducing power, with correlation coefficients (r values) ranging from 0.72 to 0.99. The TMA of GSE positively correlated with reducing power (r = 0.72*), while the TPC positively correlated with both the DPPH scavenging activity and reducing power (r = 0.97* and 0.99**, respectively). However, for RCE, only positive correlations between TPC and both the DPPH scavenging activity and reducing power were observed, with r values of 0.96** and 0.99**, respectively. Across all of the tested extracts, statistical analysis showed that only the TPC and antioxidant activities had a significantly positive correlation, with r values ranging between 0.89** and 0.90**.

### 2.3. Anthocyanin Stability of Extracts

The heat stability of aqueous extracts from BPP, GSP, and RCP was assessed at different heating temperatures (60–90 °C), heating durations (0–5 h), and pH values (2.5–5.5). The results showed that all thermal degradation data (six points for each treatment) of the anthocyanin-rich extracts fit well with a first-order reaction kinetic model, with a determination coefficient (R^2^) range of 0.83 to 0.99. The thermal degradation of anthocyanins from the BPFE, GSE, and RCE at pH 5.5 is depicted in Figure 3. The figure demonstrates that the anthocyanin retention in these aqueous extracts of various plant materials obviously decreased with increasing heating time and temperature.

Table 2 lists the kinetic degradation parameters of anthocyanins in different extracts at various heating temperatures and pH values. Generally, the rate constant (*k* value) of anthocyanins in these extracts gradually and significantly (*p* < 0.05) increased with increasing heating temperature and pH values, ranging from 0.0131 to 0.3408 h^−1^. Conversely, the half-life time (t_0.5_), which ranged from 2.03 to 52.94 h, decreased as the heating temperature and pH values increased. Among all the treatments tested, the anthocyanins in roselle calyx at 90 °C and pH 5.5 had the highest *k* value (0.3408 h^−1^) and the lowest t_0_._5_ value (2.03 h), while anthocyanins in butterfly pea at 60 °C and pH 2.5 had the lowest *k* value (0.0131 h^−1^) and the highest t_0_._5_ value (52.94 h).

High temperatures can significantly accelerate the breakdown of anthocyanin structures, leading to degradation and color loss. An increase in heating temperature from 60 to 80 °C increased the rate constant of anthocyanin degradation in BPF-containing beverages, while reducing the half-life [45]. The half-life of grape juices from various varieties, with pH values ranging from 3.32 to 3.87, was between 2.76 and 43.31 h at different heating temperatures (60–90 °C) [46]. The stability of anthocyanins is also influenced by pH, with degradation often occurring more rapidly under neutral and alkaline conditions. As the pH gradually shifts from acidic to weakly acidic, neutral, and alkaline, the structure of anthocyanins also changes, transitioning sequentially from a stable flavylium cation to an unstable carbinol pseudobase, quinoidal base, and chalcone. Anthocyanins from butterfly pea flowers appeared to have a higher stability at pH 4.0–8.0 [47], while the roselle anthocyanins degraded very quickly in a low-acid environment [9]. The half-lives of anthocyanins from the purple sweet potato extract at high temperature (90 °C) were 10.27, 12.42, and 4.66 h at pH 3.0, 5.0, and 7.0, respectively [10]. The degradation rate of anthocyanins from red onion increased nearly 17-fold as the pH rose from 1.0 to 9.0 at room temperature [23].

Under the same heating conditions, the RCE exhibited the highest rate constant and the lowest half-life time for anthocyanin degradation, whereas BPFE had the lowest rate constant and the highest half-life time. Therefore, the heat stability of anthocyanins among the three materials tested followed the descending order: butterfly pea > grape skin > roselle calyx. The results should be attributed to the types and chemical structure of anthocyanins that occurred in the plant materials. Liazid et al. indicated that the glucosyl anthocyanins were found to be more susceptible to degradation than the acylated derivatives, especially at high temperatures and in the presence of oxygen [24]. Ternatins, the main anthocyanins in BPF, are polyacylated delphinidin glucoside derivatives [14]. Black and bluish-red grape skins are rich in malvidin-3-O-glucoside and its monoacylated forms [20,21]. Natural roselle flowers are rich in sambubiosides of both delphinidin and cyanidin [17], but acylated anthocyanins in the flowers have not been reported.

Under the heating conditions of 90 °C at pH 5.5, 90 °C at pH 4.5, and 60 °C at pH 2.5, the *k* values for the BPFE, GSE, and RCE in Table 2 were 0.0461, 0.0750, and 0.0304 h ^−1^, respectively. According to Equation (2) in the Materials and Methods section, after 2 h of heating, the anthocyanin contents should be 91.2%, 86.1%, and 94.1% of their original values. Therefore, the decreases in TMA (9.1%, 20.4%, and 7.2%), as shown in Table 1, are reasonable, considering the long extraction times for BPP, GSP, and RCP at 90 °C, 90 °C, and 60 °C, respectively.

The Arrhenius model is an empirical equation used for chemical reactions and can describe the temperature dependence of the degradation reaction rate of anthocyanins.

$$k=k_{0}e^{-\frac{Ea}{RT}}$$

where *k* is the reaction rate constant, *k_0_* is the pre-exponential factor, *Ea* is the activation energy (J/mol), *R* is the gas constant (8.314 J/(mol·K)), and *T* is the absolute temperature (K).

**Figure 4:**
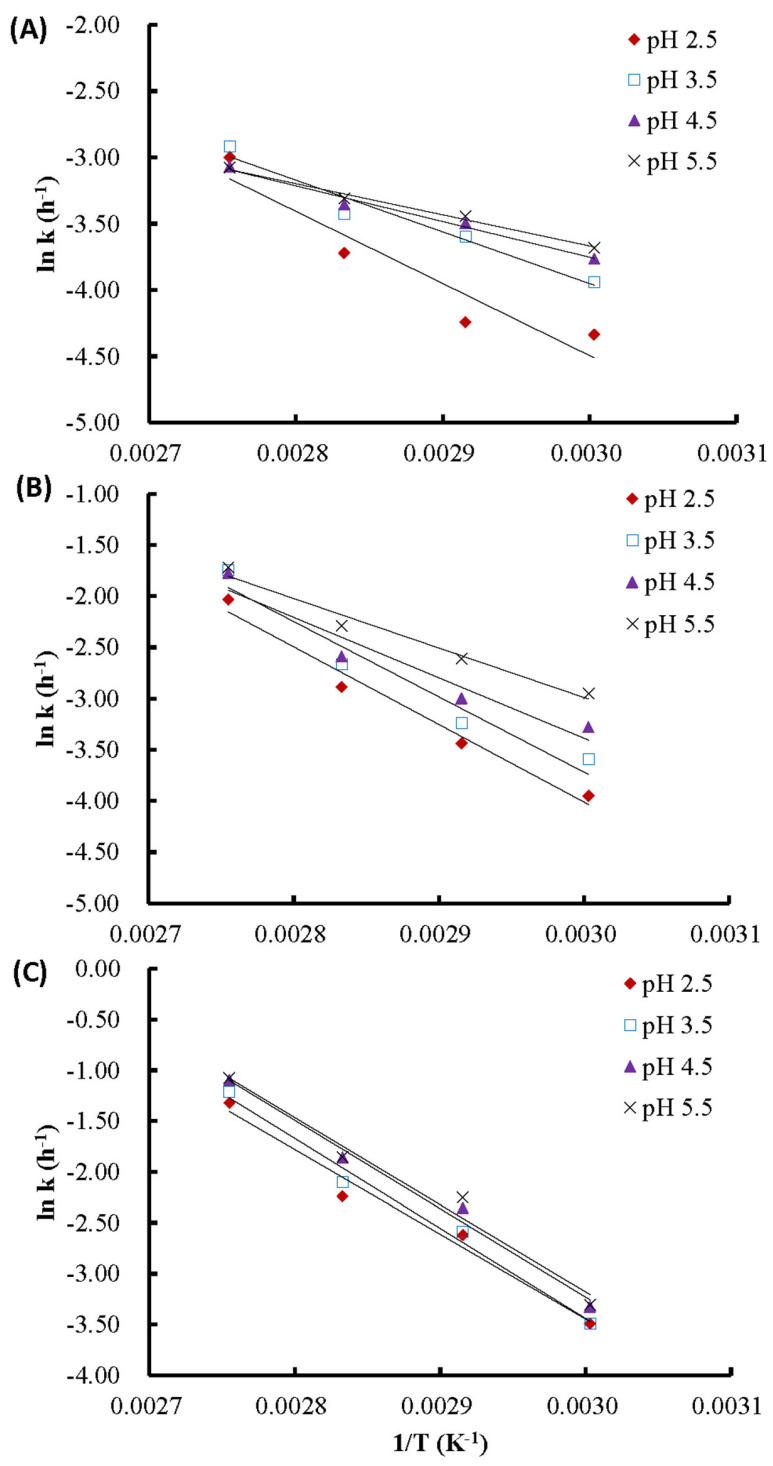
Arrhenius equation plots of anthocyanins at different pH values: (**A**) BPFE; (**B**) GSE; (**C**) RCE.

Figure 4 illustrates that the rate constants of thermal degradation are temperature-dependent and follow the Arrhenius relationship, which is in agreement with other studies [9,48]. The determination coefficients (R^2^) for BPFE, GSE, and RCE ranged from 0.90 to 0.98, 0.93 to 0.98, and 0.97 to 0.99, respectively. The activation energies for the thermal degradation of anthocyanins in BPFE at pH 2.5, 3.5, 4.5, and 5.5 were 45.23, 32.43, 22.36, and 19.52 kJ/mol, respectively. For the GSE anthocyanins, the activation energies were 63.02, 61.14, 49.07, and 40.02 kJ/mol, respectively. For the RCE anthocyanins, the activation energies were 69.25, 73.60, 72.26, and 71.29 kJ/mol, respectively.

Among the three tested materials, the order of activation energy from highest to lowest was roselle, grape skin, and butterfly pea flower. This indicates that the degradation reaction of the anthocyanins in roselle was the most sensitive to changes in reaction temperature, while the butterfly pea flower was the least sensitive. Furthermore, the activation energy of grape skin and butterfly pea flower obviously increased as the medium pH decreased, whereas the activation energy of roselle was less affected by pH. This shows that the thermal degradation reaction of anthocyanins in grape skin and butterfly pea flower was most sensitive at pH 2.5.

The activation energy of BPFE-containing beverages, formulated with different levels of sugar, salt, and ascorbic acid, ranged from 4.0 to 23.3 kJ/mol at pH 2.5 [45]. However, the activation energy for anthocyanin degradation in the BPF extract, stored at temperatures ranging from 7 °C to 90 °C and at neutral pH, varied from 83.2 to 101.2 kJ/mol [49]. For the RCE anthocyanins, the activation energies in this study were consistent with previous reports, which indicated *Ea* values for 30% ethanol and aqueous extracts from roselle ranging from 53.6 to 75.6 kJ/mol at pH 2.0–5.0 [9], 47–61 kJ/mol at pH 2.2–2.6 [32], and 66.22 kJ/mol at an unknown pH [50]. The activation energy for the GSE anthocyanins at pH 3.5 in this study was consistent with the value (64.89 kJ/mol) reported for grape juices at pH 3.34 [48], but was higher than the activation energies (44.2–45.1 kJ/mol) of grape juices from various varieties at pH 3.32–3.87 [46].

## 3. Materials and Methods

### 3.1. Materials

Ten kilograms (5 kg per batch) of fresh butterfly pea flowers (*Clitoria ternatea*) and roselle calyces (*Hibiscus sabdariffa* L.) were purchased from local farmers. Thirty kilograms of blackish-purple grapes (*Vitis vinifera* Kyoho) were purchased from a local fruit market, 10 kg per batch. GS was peeled from the grapes by hand, then immersed in water (1:20, *v*/*v*) and washed three times. The washed grape skins, BPF, and RC were dried in an oven overnight at 50 °C. Finally, the powders (i.e., BPFP, RCP, and GSP) were prepared by grinding, sifting through a 40-mesh sieve, and blending the particulates from different batches. The moisture contents of BPFP, RCP, and GSP were 9.06%, 13.20%, and 9.17%, respectively. DPPH, 6-hydroxy-2,5,7,8-tetramethylchroman-2-carboxylic acid (Trolox), ferulic acid, and sodium acetate were obtained from Sigma-Aldrich Chemical Co. (St. Louis, MO, USA). Folin–Ciocalteu phenol reagent and ferric chloride hexahydrate were obtained from Merck (Darmstadt, Germany). All chemicals used in the food analysis were of analytical grade, with a purity greater than 98%.

### 3.2. Preparation of Aqueous Extracts

Distilled water was employed as the solvent for extracting bioactive substances from BPFP, RCP, and GSP. The 2% (*w*/*v*) extracts (BPFE, RCE and GSE) were prepared in a reciprocating water bath constant temperature oscillator (Model SHZ-88A, Suzhou Peiying Experimental Equipment Co., Ltd., Suzhou, China) by mixing 4.00 g of the powder samples with 200 mL of distilled water. The extraction process lasted for different temperatures (30–90 °C) and periods (30–120 min) at 60 revolutions per minute. After extraction, the solutions were immediately filtered and cooled, then a small amount (about 4–10 mL) of distilled water was added to reach a final volume of 200 mL. Furthermore, the conventional 60% ethanol (*v*/*v*) extraction method, carried out at 40 °C for 30 min, was used as a control for comparison [34].

### 3.3. Determination of Total Monomeric Anthocyanin (TMA)

According to the pH differential method [51], the TMAs of the aqueous extracts were measured. In brief, an aliquot of 1.00 mL of the extract sample was mixed with either 4 mL of pH 1.0 buffer containing 0.025 mol/L KCl or 4 mL of pH 4.5 buffer containing 0.4 mol/L sodium acetate. The absorbance of the mixtures was determined at 520 and 700 nm wavelengths using an UV–Visible spectrophotometer (Model 752W, Shanghai Xinmao Instrument Co., Ltd., Shanghai, China). TMAs of the aqueous extracts were calculated by the following equation:

(1) $$TMA\left(\mathrm{mg}/\mathrm{kg},DB\right)=A\times F\times MW\times 1000\times V/(\varepsilon \times X\times W)$$

where A = [(A_520nm_ − A_700nm_)_pH 1_._0_ − (A_520nm_ − A_700nm_)_pH 4_._5_]; F = dilution factor; MW = 449.2 g/mol (molecular weight of cyanidin-3-glucoside); ε = 26,900 L/mol/cm (molar extinction coefficient of cyanidin-3-glucoside); x = path length (cm); V = volume of extract (mL); W = sample weight (g); DB = dry weight basis. The absorbance of each anthocyanin sample was measured three times.

### 3.4. Determination of Total Phenolics

The TPC of the aqueous extracts was measured using the Folin–Ciocalteu reagent, based on the method of [28], with ferulic acid as the standard. The TPC was reported as milligrams of ferulic acid equivalent per kilogram of sample on a dry weight basis (DB). The measurements were repeated three times.

### 3.5. Determination of Antioxidant Capacity

The radical scavenging activity of the aqueous extracts was evaluated using the DPPH assay, following the method described by Liyana-Pathirana and Shahidi [52], with Trolox as the standard. The results were expressed as milligrams of Trolox equivalents (TE) per gram of sample on a dry weight basis (DB). Additionally, the reducing power of the aqueous extracts was performed according to the method in [53]. A mixture consisting of 2.5 mL of the extract sample, 2.5 mL of 0.2 mol/L phosphate buffer (pH 6.6), and 2.5 mL of 1% potassium ferricyanide was incubated at 50 °C for 20 min. After incubation, 2.5 mL of 10% trichloroacetic acid was added, and the mixture was centrifuged at 3000 rpm for 10 min. Then, 2.5 mL of the supernatant was mixed with 2 mL of distilled water and 0.5 mL of 0.1% ferric chloride, followed by incubation at room temperature for 15 min. The absorbance was recorded at 700 nm against a blank (water), with the results expressed as mg TE per g sample (DB). The measurements of antioxidant capacity were repeated three times.

### 3.6. Determination of Anthocyanin Stability

The thermal degradation of anthocyanins in aqueous plant extracts was conducted at varying temperatures (60–90 °C), heating durations (0–5 h), and pH levels (2.5–5.5) in the water bath oscillator. The procedure involved transferring 4 mL of the aqueous extract into a glass test tube with a screw cap, followed by the addition of 4 mL of buffer (0.1 mol/L citric acid and 0.2 mol/L Na_2_HPO_4_) to adjust the pH of the sample solution to 2.5–5.5, respectively. After thorough mixing, the sample solution was heated for different periods at a specified temperature. The thermal degradation kinetics of the anthocyanins were analyzed using a first-order reaction model, expressed by Equation (2):

(2) $$\ln\left(\frac{C_{t}}{C_{0}}\right)=-k\times t$$

where *C*_0_ is the initial monomeric anthocyanin content, *C_t_* is the monomeric anthocyanin content after heating for *t* hours at a given temperature and pH, and *k* is the reaction rate constant.

The half-life time (t_0.5_), representing the time required for 50% degradation of anthocyanins, was calculated using the equation:

(3) $$t_{0.5}=\frac{ln2}{k}$$

### 3.7. Statistical Analysis

The data in triplicate or duplicate for different treatments were analyzed by one-way ANOVA and Duncan’s new multiple range test to determine the statistical significance of differences among the values using IBM SPSS Statistics 20.

## 4. Conclusions

In this study, 2% (*w*/*v*) aqueous extracts of butterfly pea flower, grape skin, and roselle calyx were prepared. To maximize the total anthocyanins and polyphenols in these extracts, powders of BPF, GS, and RC were extracted for 30 min at 90 °C; 30 min and 120 min at 90 °C; and 30 min and 60 min at 60 °C, respectively. The TMA values of the plant materials, in descending order, were RCE (4.04 mg/g), GSE (2.79 mg/g), and BPFE (1.75 mg/g), while the TPC (mg FAE/g) followed the order: GSE (69.2) > BPFE (30.0) > RCE (25.4). Among the tested plant materials, BPF exhibited the lowest antioxidant activities (radical DPPH scavenging activity and reducing power), likely due to having fewer hydroxyl groups on the B-ring of its anthocyanin structure. The correlations between TPC and antioxidant activities were stronger than those between TMA and antioxidant activities.

The thermal degradation of anthocyanins from the plant materials followed a first-order reaction model. All rate constants of thermal degradation for BPE, GSE, and RCE increased with rising heating temperatures. However, the half-life of the extracts decreased as the temperature increased. BPE, GSE, and RCE exhibited better heat stability of the anthocyanins at lower pH levels. The temperature-dependent rate constants followed the Arrhenius relationship. The activation energy of the three extracts in decreasing order was: RCE > GSE > BPE. The *Ea* values for BPE and GSE decreased with an increase in the pH of the extract. Butterfly pea flower had the highest heat stability of anthocyanins, while roselle calyx had the least heat stability. The results may be attributed to the degree of acylation in the anthocyanin structures in the various plant materials. Finally, this study confirms that hot water extraction is feasible for butterfly pea flower, roselle calyx, and grape skin. Under appropriate extraction conditions of temperature and time, the resulting extracts are rich in anthocyanins and total polyphenols, with high antioxidant capacity, and are convenient and safe to apply in food products such as yogurts, jellies, confectioneries, noodles, and functional beverages [6,7,28].

## Figures and Tables

**Table 2:** Thermal degradation parameters of anthocyanins in various extracts at different heating temperatures and pH.

pH	Heating	BPFE	BPFE	GSE	GSE	RCE	RCE
	Temperature (°C)	*k* × 100 (h^−1^)	t_0.5_ (h)	*k* × 100 (h^−1^)	t_0.5_ (h)	*k* × 100 (h^−1^)	t_0_._5_ (h)
2.5	60	1.31 ± 0.05 ^h^	52.94 ± 2.41 ^a^	1.93 ± 0.10 ^i^	35.91 ± 1.82 ^a^	3.04 ± 0.14 ^g^	22.80 ± 1.15 ^a^
2.5	70	1.44 ± 0.06 ^h^	48.16 ± 2.08 ^b^	3.22 ± 0.19 ^hi^	21.53 ± ^1.30 c^	7.29 ± 0.45 ^f^	9.51 ± 0.57 ^c^
2.5	80	2.42 ± 0.21 ^fg^	28.64 ± 2.47 ^d^	5.58 ± 0.36 ^ef^	12.42 ± 0.82 ^e^	10.64 ± 0.74 ^e^	6.51 ± 0.43 ^de^
2.5	90	5.00 ± 0.46 ^ab^	13.87 ± 1.18 ^i^	13.09 ± 1.03 ^b^	5.30 ± 0.42 ^gh^	26.70 ± 2.12 ^c^	2.60 ± 0.21 ^g^
3.5	60	1.95 ± 0.12 ^gh^	35.56 ± 2.51 ^c^	2.75 ± 0.12 ^hi^	25.21 ± 1.17 ^b^	3.05 ± 0.14 ^g^	22.73 ± 1.06 ^a^
3.5	70	2.74 ± 0.28 ^def^	25.37 ± 2.80 ^def^	3.91 ± 0.24 ^gh^	17.73 ± 1.10 ^d^	7.50 ± 0.46 ^f^	9.24 ± 0.57 ^c^
3.5	80	3.26 ± 0.39 ^cd^	21.25 ± 2.74 ^fg^	6.97 ± 0.45 ^de^	9.94 ± 0.65 ^f^	12.22 ± 0.79 ^e^	5.67 ± 0.36 ^ef^
3.5	90	5.41 ± 0.59 ^a^	12.87 ± 1.34 ^i^	17.44 ± 1.42 ^a^	3.97 ± 0.32 ^h^	29.80 ± 2.45 ^b^	2.33 ± 0.19 ^g^
4.5	60	2.32 ± 0.24 ^fg^	29.88 ± 3.12 ^d^	3.78 ± 0.14 ^gh^	18.34 ± 0.67 ^d^	3.60 ± 0.12 ^g^	19.25 ± 0.70 ^b^
4.5	70	3.04 ± 0.32 ^cdef^	22.80 ± 2.47 ^efg^	4.99 ± 0.24 ^fg^	13.89 ± 0.66 ^e^	9.49 ± 0.45 ^ef^	7.30 ± 0.35 ^d^
4.5	80	3.49 ± 0.35 ^c^	19.86 ± 2.18 ^g^	7.50 ± 0.44 ^d^	9.24 ± 0.62 ^f^	15.61 ± 1.04 ^d^	4.44 ± 0.29 ^f^
4.5	90	4.66 ± 0.35 ^b^	14.91 ± 1.12 ^hi^	16.99 ± 1.30 ^a^	4.08 ± 0.31 ^h^	33.41 ± 2.70 ^a^	2.07 ± 0.15 ^g^
5.5	60	2.52 ± 0.15 ^efg^	27.51 ± 2.12 ^de^	5.22 ± 0.26 ^fg^	13.28 ± 0.67 ^e^	3.67 ± 0.18 ^g^	18.89 ± 0.96 ^b^
5.5	70	3.20 ± 0.19 ^cde^	21.68 ± 1.35 ^fg^	7.35 ± 0.43 ^d^	9.43 ± 0.56 ^f^	10.53 ± 0.63 ^e^	6.58 ± 0.39 ^de^
5.5	80	3.66 ± 0.32 ^c^	18.97 ± 1.65 ^gh^	10.15 ± 0.67 ^c^	6.83 ± 0.45 ^g^	15.64 ± 1.04 ^d^	4.43 ± 0.29 ^f^
5.5	90	4.61 ± 0.31 ^b^	15.07 ± 1.01 ^hi^	17.87 ± 1.41 ^a^	3.88 ± 0.31 ^h^	34.08 ± 2.70 ^a^	2.03 ± 0.15 ^g^

Values are the means ± standard deviations (*n* = 2), and means with different letters in the same column are significantly different (*p* < 0.05). BPFE: butterfly pea flower extract; GSE: grape skin extract; RCE: roselle calyx extract; *k*: rate constant; t_0.5_: half-time.

## Data Availability

The original contributions presented in the study are included in the article, further inquiries can be directed to the corresponding author/s.
